# Development of the Scores for Traditional and Modified Japanese Diets

**DOI:** 10.3390/nu15143146

**Published:** 2023-07-14

**Authors:** Haruka Miyake, Ikuko Kashino, Akiko Nanri, Tetsuya Mizoue

**Affiliations:** 1 Department of Epidemiology and Prevention, Center for Clinical Sciences, National Center for Global Health and Medicine, Tokyo 162-8955, Japanmizoue@hosp.ncgm.go.jp (T.M.); 2 Section of Research of Collaboration and Partnership, National Institutes of Biomedical Innovation, Health and Nutrition, Osaka 566-0002, Japan; 3 Department of Food and Health Sciences, International College of Arts and Sciences, Fukuoka Women’s University, Fukuoka 813-8529, Japan

**Keywords:** Japanese diet, diet score, diet quality, Japan

## Abstract

Increasing attention is being paid to the role of diet quality in the prevention and management of non-communicable diseases. We developed a scoring system for the traditional Japanese diet and its modified version considering the dietary culture in Japan, dietary guidelines for the Japanese, and updated evidence for disease prevention. The traditional Japanese diet comprises white rice, miso soup, soybean products, vegetables, mushrooms, seaweeds, fish and shellfish, high-sodium foods, and green tea. In the modified Japanese diet, unprocessed or minimally refined rice and raw vegetables, milk and dairy products, and fruits were additionally considered, while salty food was reverse-scored. The cutoff for the intake frequency of each food/food group was determined with reference to a health survey of >12,000 workers. Among the participants in the validation study, we confirmed the nutritional gradient with increasing scores in the expected direction. The scores were closely correlated with the respondents’ backgrounds, including occupational factors. This simple scoring system can be used for diet quality assessments and epidemiological research.

## 1. Introduction

Increasing attention is being paid to the role of overall diet quality in the prevention and management of non-communicable diseases. Several dietary quality indices have been developed and are linked to the risk of mortality and morbidity. Based on a systematic review and meta-analysis of 113 reports from cohort studies, Morze et al. [1] reported a lower risk of all-cause mortality, cardiovascular disease (CVD), cancer, type 2 diabetes, and neurodegenerative diseases among those with the highest diet quality, as assessed using the Healthy Eating Index, Alternate Healthy Eating Index, and Dietary Approaches to Stop Hypertension scores, as well as a lower risk of all-cause and cancer mortality among cancer survivors. As diet is an important component of culture, it is desirable to consider local diets when creating a diet score tailored to each country or region.

The traditional Japanese diet (*Washoku*) is characterized by white rice, miso soup, fish, vegetables, mushrooms, soybean products, and seaweed. The unique variability in *Washoku* may reflect the climatic or geographical features of Japan [2]. For example, the climate (four seasons, temperate zones, and high rainfall) is suitable for the growth of rice, vegetables, and mushrooms. Fresh fish and seaweed are available in Japan, which is surrounded by the sea. Moreover, heavy rain and soft water may have prompted the development of various cooking methods, including steaming, boiling, and simmering, which have been applied to the production of steamed rice, miso soup, boiled vegetables, and soybean products such as tofu. Similarly, fermented foods, including miso, natto, soy sauce, and pickled vegetables, have been developed because of the hot and humid summers in Japan.

Several dietary scoring systems [3,4,5,6,7,8] have been developed in Japan and their associations with health status have been investigated. Adherence to the Japanese Food Guide Spinning Top (JFGSP) [3] has been linked to a lower risk or prevalence of depression [9], metabolic risk factors [10,11,12], frailty [13], and mortality [14,15]. However, this score measures adherence to a healthy diet and is not specific to the Japanese diet. Several scores have been developed to measure adherence to the Japanese-style diet. The Japanese Diet Index (JDI) [5,6], Japanese Healthy Diet Index (JHDI) [7], Japanese Food Score (JFS) [8] and their higher scores have been associated with a lower risk of dementia (JDI) [6], functional disability [5,16,17] (JDI), mortality (JDI) [18], metabolic risk factors (JHDI) [7], and all-cause and CVD mortality (JFS) [8], respectively. Reduced-Salt Japanese Diet Score (RSJDS) has been linked to a low risk of all-cause mortality, CVD, and stroke [4].

While the Japanese diet is thought to be healthy in terms of vascular health (low in saturated fat and high in n-3 polyunsaturated fat) and might contribute to longevity [19], nutritional inadequacy among the Japanese has also been documented, including a high sodium intake and insufficient intake of calcium and dietary fiber [20]. These issues have not been well addressed in the scoring of the Japanese-style diet. From a public health nutrition perspective, there is a need to create a new diet score that considers these issues. Thus, the objective of the present study was to develop a simple score of diet quality for the Japanese population, which would help in dietary assessment and education and facilitate nutritional epidemiological studies for the prevention of chronic diseases.

## 2. Methods

### 2.1. Overview

We previously developed and validated a short food frequency questionnaire (FFQ) designed to assess usual diet, including typical Japanese foods [21]. Based on this questionnaire, we developed scores for traditional and modified Japanese diets. As a reference to determine the cutoff of each food item for scoring, we examined the distribution of food intake frequency among participants in a large epidemiological survey in which the above diet questionnaire was incorporated. Among the validation study participants, we assessed the correlation between these scores and nutrient intake. Finally, we calculated the scores for each participant in the working population study and examined these scores in relation to their backgrounds.

### 2.2. Dietary Questionnaire

We created a 28-item FFQ [21], which comprises 21 food groups (rice with barley and millet, brown rice, and rice with germ; white rice; whole-grain bread, rye bread, barley bread, and millet bread; other bread such as white bread and sweet buns; noodles; potatoes; miso soup; soy products; raw vegetables; cooked vegetables; mushrooms; seaweeds; fruits; fish and shellfish excluding dried fish and salty fish; beef, pork, liver, and processed meat; chicken; eggs; milk and dairy products; nuts; salty foods such as pickled plums, pickled vegetables, dried fish, salty fish, and fish roe; fried foods such as tempura, fried chicken, deep-fried foods, cutlets, and French fries) and 7 beverage items (coffee; green tea; other tea such as black tea, oolong tea, and blend tea; water; 100% fruit and vegetable juice; sugar-sweetened beverages including soft drinks, coffee, black tea; artificially sweetened beverages such as non-caloric and low-caloric beverages). For each item, we prepared eight options regarding consumption frequency over the past month (ranging from never to ≥3 times/day for food groups and from never to ≥5 cups/day for beverage items). According to the validation study using a 3-day photographic dietary record as the gold standard (described later), the Spearman correlation coefficient between intake frequency and crude intake of food groups ranged from −0.12 to 0.86 (median 0.51). The corresponding values for energy-adjusted intake using the residual methods [22] ranged from −0.01 to 0.82 (median 0.47).

### 2.3. Pointing System

Table 1 shows the methods used to develop the dietary scores for the Japanese population. For the traditional Japanese diet, we selected nine food groups: white rice, miso soup, soybean products, cooked vegetables, mushrooms, seaweed, fish, shellfish, high-sodium foods, and green tea. For the modified Japanese diet with a better nutritional profile, we replaced white rice with whole grains (i.e., rice mixed with millet and barley, brown rice, germinated rice, under- or half-milled rice), included cooked vegetables, raw vegetables and 100% vegetable and fruit juice as “vegetables”, and added fruit and milk and dairy products, while we reversed the scoring for high-sodium foods (total of11 items). For vegetables, we assigned the mid-value of intake frequency (converted into per day) for each component (0—hardly consumed; 0.07—1–3 times/month; 0.21—1–2 times/week; 0.5—3–4 times/week; 0.79—5–6 times/week; 1–1 times/day; 2–2 times/day; and 3–3 times/day or more), doubled the value for cooked vegetables and halved the value for 100% vegetable and fruit juice, and then summed them to calculate the total intake.

According to the worker survey data (described later), we determined the cutoff value based on the median intake (frequency or service) of each food or food group. One point was given to each food/food group that met the cutoff value (above or below, depending on the item). For whole grains, for which the median intake was less than three times per week, we set the cut-off at three times/week.

### 2.4. Assessment of the Diet Scores

#### 2.4.1. Worker Survey

As part of the Japan Epidemiology Collaboration on Occupational Health Study (J-ECOH) [23,24], an ongoing epidemiological study examining the health determinants of Japanese workers, a health survey including the above diet questionnaire was performed in 2018–2021 in five participating companies [25], which also provided information on the results of a health check-up. Of the 12,847 participants in the questionnaire survey (response rate: 75.2%), we excluded those without information on the frequency of food item intake (*n* = 87). Consequently, data from 12,760 participants (11,212 men and 1548 women, aged 18–80) were used to assess the distribution of food intake and to examine the diet scores in relation to the sociodemographic factors. The study protocol was approved by the Ethics Committee of the National Center for Global Health and Medicine (NCGM-S-001140-20).

#### 2.4.2. Validation Study

A validation study of the above-mentioned short FFQ was conducted among 24 faculty members of Fukuoka Women’s University (9 men and 15 women, aged 23–64 years) [21]. The participants were asked to take a picture of everything they ate and drank for three consecutive days (photographic food records) and bring them to a laboratory to confirm what they consumed the day following the last day of photographic food recording (interview survey). The number of days for photographic food records was determined to cover the diet on both weekdays and weekends while minimizing the burden on the participants. The participants were also asked to answer the FFQ that we developed twice (Time 1 and Time 2). The participants responded to the paper-based FFQ (Time 1) in the laboratory before the interview survey. They then received the FFQ (Time 2) as an e-mail attachment one week later and submitted their completed FFQ (Time 2) via e-mail or by hand with the printed-out paper version. The study protocol was approved by the Ethics Committee of Fukuoka Women’s University (2018–2020), and written informed consent was obtained from all participants prior to the survey.

### 2.5. Statistical Analysis

Descriptive data were presented as means and standard deviations for continuous variables or numbers and percentages for categorical variables. Data from the worker survey were used to characterize the diet scores in relation to the sociodemographic factors. We divided the participants into the quartiles of each diet score and examine the association with socio-demographic factors: age (years, continuous), sex (men or women), educational background (9–12 years, 13–16 years, or ≥17 years), employment status (full-time or others), job rank (upper, middle, or lower), work shift (day-time only, night or rotating shift, or others), occupation (white-collar, blue-collar, or not classified), marital status (yes or no), hours of sleep (<6 h/day, 6 to 6.9 h/day, or ≥7 h/day), current smoking status (yes or no), alcohol consumption (non-drinker, drinker consuming <1 *go*/day, 1–1.9 *go*/day, or ≥2 *go*/day), and leisure-time exercises (no exercise, <3 metabolic equivalents [METs], 3 to 9.9 METs, or ≥10 METs). The body mass index (BMI) was calculated as body weight divided by the height squared based on the results of a health check-up and categorized (<18.5 kg/m^2^, 18.5 to 24.9 kg/m^2^, or ≥25 kg/m^2^). The above analyses were performed using Stata, version 16.0 (StataCorp, College Station, TX, USA).

The data from the validation study were used to examine the nutritional aspects of the newly developed diet scores. We calculated the traditional and modified Japanese diet scores based on the FFQ (Time 1). We also calculated the average daily intake of 28 food groups and beverages in the FFQ, energy, and nutrients based on a 3-day photographic dietary record with reference to the Standard Tables of Food Composition in Japan [26]. The intakes of food groups and nutrients were adjusted for energy consumption using the residual method [22]. Spearman’s correlation coefficients were calculated between the diet scores and the crude intake or energy-adjusted intake of food groups and nutrients derived from photographic food records. The analyses were performed using SAS version 9.4 (SAS Institute Inc., Cary, NC, USA).

## 3. Results

### 3.1. Distribution of Diet Score

**Figure 1:**
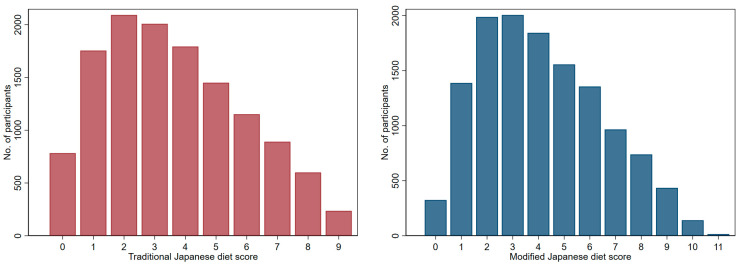
The distribution of study participants for each score developed.

Both scores showed a right-skewed distribution (Figure 1). The mean (standard deviation) and mode of traditional Japanese diet score were 3.6 (2.3) and 2, respectively. Those of the modified Japanese diet score were 4.1 (2.4) and 3, respectively. The percentage of people who achieved the cutoff values for each component food/food group showed increasing trends with increasing scores for both diets, except for high-sodium foods in relation to the modified Japanese diet (Supplementary Tables S1 and S2)

### 3.2. Association with Background Factors

Table 2 shows the background factors of the study participants in relation to the dietary scores. Female sex, older age, higher educational attainment, higher levels of leisure-time physical activity, higher job rank, white-collar occupations, and being married were each associated with a higher score of the traditional Japanese diet score, whereas full-time employment and current smoking were each associated with a lower score. The score did not differ much according to sleeping hours, alcohol consumption, and BMI. The associations (especially, those with sex, occupation, and smoking) were more pronounced for the modified Japanese diet score.

### 3.3. Association with Dietary Intake

Table 3 shows the correlation between diet scores and the intake of each component based on the 3-day photographic food record. Spearman correlation coefficients between the diet quality scores and crude intakes of each component estimated from the record ranged from 0.12 (white rice) to 0.49 (miso soup) for the traditional Japanese diet score and ranged from 0.14 (cooked vegetables) to 0.42 (fruits) for the modified Japanese diet score. The values were 0.20–0.50 for components other than white rice, cooked vegetables, and green tea in the traditional Japanese diet score and cooked vegetables in the modified Japanese diet score (<0.20). Energy adjustments did not substantially change the results.

Table 4 shows spearman correlation coefficients between diet scores and nutrient intakes estimated from the food record. Both scores were positively associated with intakes of protein, n-3 PUFA, carbohydrate, dietary fiber, sodium, potassium, calcium, magnesium, iron, vitamin A, D, E, and C, and folate, but inversely associated with intakes of fat, saturated fatty acids, and n-6 PUFA. Relatively high correlations between the diet scores and crude intakes of nutrients (>0.4) were observed for potassium, calcium, magnesium, iron, vitamin D, folate, and vitamin C. After the adjustment of energy intake, the correlation coefficients with the intakes of some nutrients were strengthened.

## 4. Discussion

We developed two scores that measured the adherence to the Japanese diet based on a short food group-based diet questionnaire: one for the traditional diet and the other for its modified version. Both scores showed moderate correlations with food and nutrient intake in the expected direction in a sample of university personnel, as well as close associations with participants’ backgrounds in a large working population.

### 4.1. Comparison with Previous Scores for Japanese

Several dietary scoring systems have been developed to assess the diet quality in the Japanese population (Supplementary Table S3). The JFGSP was intended to promote a healthy diet among the Japanese people [3]. The original JFGST consists of six food groups (grain dishes, vegetable dishes, fish and meat dishes, dairy, fruits, energy from snacks, and alcoholic beverages) [3,14], whereas the modified versions also includes the white/red meat ratio [13,15,27] and sodium [9,28]. Dietary scores emphasizing local foods in Japan have also been proposed. The RSJDS was developed based on seven dietary components associated with the risk of mortality (intake of Japanese pickles, eggs, fish, meat, soup with noodles, use of low-salt soy sauce, and occasional drinking) [4]. The JDI [5,6], JHDI [7], and JFS [8] were established based on dietary patterns identified using factor analysis to characterize the traditional Japanese diet. The JDI scored seven positive (rice, miso soup, green and yellow vegetables, seaweeds, pickled vegetables, fish, and green tea) and two negative food items (beef and pork and coffee) [5,6]. The JHDI contains 10 positive-scored items (rice, boiled beans, tofu, fermented beans, miso soup, white vegetables, green vegetables, red/yellow vegetables, fruits, and fish) and 9 reverse-scored items (bread, soybean milk, vegetable juice, fruit juice, beef/pork, chicken, ham, milk, and yoghurt) [7]. The JFS consists of seven foods (beans and bean products, fresh fish, vegetables, Japanese pickles, fungi, seaweed, and fruits) [8]. The procedure for our pointing system resembles that used in previous Japanese studies. Specifically, one point was given to each food item if the intake frequency/serving of a food/food group characterizing the diet was equal to or above the median intake (JDI, JHDI), summing the points of all the component food items to obtain the total score.

### 4.2. Association of the Diet Scores with Participants’ Characteristics

In our large working population survey, higher scores on the traditional and modified Japanese diets were associated with greater odds of being female, older, married, physically active, non-smokers, and having higher educational attainment, but they were not associated with alcohol consumption and BMI. Some of these associations have been previously reported. For instance, Tomata et al. found associations between higher JDI scores and female sex, higher educational attainment, engagement in walking, and non-smoking [5,6], while Okada et al. identified associations between higher JFS scores and being older, more educated, engaged in longer hours of sports activity, and non-smoking [8]. Regarding occupational factors, we found that white-collar and higher rank occupations, and employment other than full-time were associated with higher Japanese diet scores. These factors can be regarded as sociodemographic correlates of the Japanese diet and may thus play a role as potential confounders or mediators that need to be adequately addressed in epidemiological investigations of the relationship between the Japanese diet and health outcomes. As this analysis was carried out among predominantly male workers in Kanto and other specific regions in Honshu (the main island of Japan), the associations may differ in a population with a different background or in a different region.

### 4.3. Association of the Developed Scores with Intakes of Foods and Nutrients

Moderate correlations were observed between the traditional Japanese diet score and food intake estimated from food records, except for white rice (*r* = 0.12) and cooked vegetables (*r* = 0.19). This might reflect the small variation in the intake of these foods among the participants in the validation study. Specifically, the mean intake of white rice was 204 g/day and 258 g/day for participants below the cutoff and those equal to or above the cutoff, respectively. The corresponding values of cooked vegetables in the modified Japanese diet were 113 g/day and 135 g/day, respectively.

In the present study, both diet scores were significantly and positively associated with intakes of potassium, calcium, magnesium, iron, vitamin D, folate, and vitamin C. Zhang et al. [29] reported relatively high positive correlations (>0.4) of the scores of the 12-component modified JDI (similar to the traditional Japanese diet) with the intake of dietary fiber, potassium, magnesium, iron, and vitamin C, and an inverse correlation with saturated fat intake. We also observed relatively strong correlations with these nutrients except for dietary fiber.

### 4.4. Conclusions

Based on a 28-item FFQ, we developed a scoring system that measures the adherence to the traditional Japanese diet and a modified version with a better nutritional profile. These scores moderately correlated with the intake of relevant foods and nutrients and were closely associated with demographic, socioeconomic, occupational, and lifestyle factors among workers. The short diet questionnaire and simple scoring system may suit nutritional assessment in a resource-limited setting and facilitate nutritional epidemiological studies on the association between the Japanese diet and various health outcomes.

## Figures and Tables

**Table 1:** The components and cutoff values for scores developed.

	Traditional Japanese Diet Score	Modified Japanese Diet Score
Food/Food Group	Cut off ^1^	Cut off ^1^
White rice	≥2 times/day	-
Whole grains	-	≥3 times/week ^2^
Miso soup	≥5 times/week	≥5 times/week
Soybean products	≥3 times/week	≥3 times/week
Vegetables	≥5 times/week ^3^	≥1.7 servings/day ^4^
Mushrooms	≥3 times/week	≥3 times/week
Seaweeds	≥3 times/week	≥3 times/week
Fruits	-	≥3 times/week
Fish and shellfish	≥3 times/week	≥3 times/week
Milk and dairy products	-	≥5 times/week
High-sodium foods ^5^	≥1 time/week	<1 time/week
Green tea	≥1 cup/day	≥1 cup/day
Total score (points)	9	11

^1^ The cutoff value was determined based on the median frequency (or serving) of each component intake, except for whole grains (≥3 times/week for the modified Japanese diet score). One point was assigned to each component that meets the cutoff value. ^2^ Including rice mixed with millet and barley, brown rice, germinated rice, under- or half-milled rice. ^3^ Including only cooked vegetables (e.g., dishes with lots of vegetables and soups). ^4^ Including cooked vegetables, raw vegetables, and 100% fruits and vegetable juice. ^5^ Including pickled plums, pickled vegetables, dried fish, salted fish, and fish roe.

**Table 2:** Characteristics of study participants according to the quartiles of the diet scores.

	Traditional Japanese Diet Score				Modified Japanese Diet Score			
	Q1 (n = 4630)		Q4 (n = 2879)		Q1 (n = 3701)		Q4 (n = 2296)	
Characteristics								
Age, mean [SD]	39.7	[12.1]	45.1	[12.5]	39.5	[12.0]	45.7	[12.2]
Sex (female)	506	(10.9)	372	(12.9)	311	(8.4)	375	(16.3)
Educational attainment								
9–12 years	2447	(52.9)	1286	(44.7)	2164	(58.6)	941	(41.1)
13–16 years	1299	(28.1)	787	(27.4)	933	(25.3)	647	(28.2)
≥17 years	878	(19.0)	802	(27.9)	597	(16.2)	704	(30.7)
Employment status								
Full-time	4259	(92.1)	2492	(86.6)	3402	(92.0)	1984	(86.4)
Others	367	(7.9)	386	(13.4)	296	(8.0)	311	(13.6)
Job rank								
Upper	141	(3.1)	188	(6.5)	107	(2.9)	157	(6.8)
Middle	1062	(23.0)	909	(31.6)	776	(21.0)	762	(33.2)
Lower	3419	(74.0)	1779	(61.9)	2813	(76.1)	1373	(59.9)
Shift work								
Daytime	3187	(68.9)	2183	(75.8)	2444	(66.1)	1770	(77.1)
Night and/or rotating shift	1068	(23.1)	448	(15.6)	954	(25.8)	305	(13.3)
Others	373	(8.1)	248	(8.6)	301	(8.1)	221	(9.6)
Occupation								
White-collar	2454	(53.2)	1813	(63.1)	1795	(48.6)	1533	(66.9)
Blue-collar	1985	(43.0)	924	(32.2)	1757	(47.6)	652	(28.5)
Not classified	177	(3.8)	136	(4.7)	139	(3.8)	106	(4.6)
Marital status								
Married	2546	(55.0)	2095	(72.9)	2064	(55.8)	1687	(73.6)
Not married	2081	(45.0)	780	(27.1)	1633	(44.2)	605	(26.4)
Daily sleeping hours ^1^								
<6 h	1211	(26.2)	738	(25.7)	953	(25.8)	611	(26.6)
6–6.9 h	1820	(39.3)	1163	(40.4)	1438	(38.9)	944	(41.2)
≥7 h	1595	(34.5)	976	(33.9)	1307	(35.3)	739	(32.2)
Smoking status								
Non-smoker	3125	(67.5)	2265	(78.7)	2387	(64.5)	1874	(81.6)
Current smoker	1504	(32.5)	614	(21.3)	1311	(35.5)	422	(18.4)
Daily alcohol consumption								
Non-drinker	1209	(26.1)	704	(24.5)	907	(24.7)	632	(27.5)
<1 *go* ^2^	2238	(48.4)	1407	(48.9)	1766	(47.7)	1115	(48.6)
1–1.9 *go* ^2^	712	(15.4)	492	(17.1)	593	(16.0)	368	(16.0)
≥2 *go* ^2^	469	(10.1)	273	(9.5)	434	(11.7)	180	(7.8)
Body mass index								
<18.5 kg/m^2^	214	(4.7)	128	(4.5)	176	(4.8)	102	(4.5)
18.5–24.9 kg/m^2^	3056	(66.9)	1896	(66.7)	2381	(65.4)	1516	(67.0)
≥25 kg/m^2^	1296	(28.4)	819	(28.8)	1081	(29.7)	645	(28.5)
Weekly leisure time exercise								
No exercise	1328	(28.7)	431	(15.0)	1160	(31.4)	288	(12.6)
<3 METs	1313	(28.4)	798	(27.8)	1059	(28.6)	639	(27.9)
3–9.9 METs	927	(20.0)	715	(24.9)	704	(19.0)	582	(25.4)
≥10 METs	1057	(22.9)	931	(32.4)	774	(20.9)	785	(34.2)

^1^ On weekday ^2^ *go*: the standard unit (180 mL) of the volume of sake. One *go* of sake contains 23g ethanol. Respondents were asked to answer their alcohol consumption in sake-equivalent volume according to the ethanol concentrations of each beverage. Due to missing values, the total number of participants across all the categories of a given variable is not equal to the number of participants for each quartile of dietary pattern. The total number of missing values ranges between 3 (smoking) and 177 (body mass index). Figures are numbers (percentages) unless otherwise stated. Abbreviations: Q: quartile; SD: standard deviation; METSs: metabolic equivalents.

**Table 3:** Spearman correlation coefficients between the traditional and modified Japanese diet scores and Intake of each component of the dietary scores based on 3-day photographic food record (n = 24).

	**Traditional Japanese Diet Score**				**Modified Japanese Diet Score**			
	**Crude**		**Energy-Adjusted ^1^**		**Crude**		**Energy-Adjusted ^1^**	
	**r**	***p***	**r**	***p***	**r**	***p***	**r**	***p***
White rice	0.12	0.56	0.06	0.77	-	-	-	-
Whole grains	-	-	-	-	0.34	0.11	0.29	0.18
Miso soup	0.49	0.016	0.43	0.038	0.31	0.14	0.28	0.18
Soybean products	0.34	0.10	0.17	0.42	0.29	0.17	0.13	0.55
Raw vegetables	-	-	-	-	0.25	0.23	0.10	0.64
Cooked vegetables	0.19	0.38	0.22	0.30	0.14	0.52	0.16	0.46
100% fruits and vegetable juice	-	-	-	-	0.20	0.34	0.09	0.66
Mushrooms	0.23	0.27	0.24	0.25	0.35	0.090	0.38	0.070
Seaweeds	0.37	0.076	0.40	0.053	0.32	0.13	0.34	0.10
Fruits	-	-	-	-	0.42	0.040	0.43	0.038
Fish and shellfish	0.41	0.045	0.39	0.063	0.40	0.051	0.36	0.085
Milk, dairy products	-	-	-	-	0.31	0.14	0.29	0.17
High-sodium foods	0.37	0.073	0.39	0.059	0.22	0.30	0.22	0.29
Green tea	0.19	0.38	0.23	0.28	0.25	0.24	0.32	0.13

^1^ Food group intakes based on photographic food records were adjusted for energy intake by the residual method.

**Table 4:** Spearman correlation coefficients between the traditional and modified Japanese diet scores and nutrient intakes based on 3-day photographic food record (n = 24).

	Traditional Japanese Diet Score				Modified Japanese Diet Score			
	Crude		Energy-Adjusted ^1^		Crude		Energy-Adjusted ^1^	
	r	*p*	r	*p*	r	*p*	r	*p*
Protein (g)	0.15	0.49	0.20	0.35	0.19	0.37	0.23	0.28
Fat (g)	−0.27	0.21	−0.47	0.022	−0.14	0.51	−0.28	0.19
Saturated fatty acids (g)	−0.17	0.42	−0.35	0.095	−0.05	0.81	−0.20	0.35
n-3 PUFA (g)	0.14	0.53	0.16	0.46	0.16	0.47	0.14	0.50
n-6 PUFA (g)	−0.14	0.52	−0.31	0.15	−0.08	0.70	−0.22	0.30
Carbohydrate (g)	0.22	0.31	0.54	0.006	0.19	0.38	0.40	0.052
Total dietary fiber (g)	0.33	0.11	0.55	0.006	0.35	0.094	0.48	0.017
Sodium (mg)	0.24	0.27	0.31	0.14	0.18	0.39	0.10	0.63
Potassium (mg)	0.52	0.009	0.66	0.001	0.55	0.005	0.67	<0.001
Calcium (mg)	0.53	0.008	0.53	0.007	0.54	0.006	0.54	0.007
Magnesium (mg)	0.57	0.004	0.67	<0.001	0.55	0.005	0.60	0.002
Iron (mg)	0.59	0.002	0.70	<0.001	0.56	0.004	0.65	0.001
Vitamin A (µg)	0.34	0.11	0.24	0.26	0.32	0.13	0.18	0.39
Vitamin D (µg)	0.47	0.019	0.48	0.019	0.42	0.040	0.41	0.047
Vitamin E (mg) ^2^	0.25	0.23	0.35	0.093	0.29	0.16	0.39	0.057
Folate (µg)	0.51	0.011	0.48	0.018	0.54	0.006	0.48	0.018
Vitamin C (mg)	0.45	0.029	0.46	0.025	0.51	0.011	0.50	0.012

^1^ Nutrient intakes based on photographic food records were adjusted for energy intake by the residual method. ^2^ α-tocopherol.

## Data Availability

The data are not publicly available but are available upon reasonable request to the corresponding author.

## Supplementary Materials

The following supporting information can be downloaded at: https://www.mdpi.com/article/10.3390/nu15143146/s1, Supplementary Table S1: Number (percentage) of people who achieved the cutoff for each component food/food group according to the quartiles of the traditional Japanese diet score; Supplementary Table S2: Number (percentage) of people who achieved the cutoff for each component food/food group according to the quartiles of the modified Japanese diet score; Supplementary Table S3: Summary of existing scoring systems of Japanese diet.
