# Supplementary material for: Development of the Scores for Traditional and Modified Japanese Diets
Source: Nutrients. 2023 Jul 14;15(14):3146. doi: 10.3390/nu15143146 (PMC10386055; doi:10.3390/nu15143146)
Supplement: Supplementary file 1 [file nutrients-15-03146-s001.zip › nutrients-2463612-supplementary.pdf]

Supplementary Table S1. Number (percentage) of people who achieved the cutoff for each component food/food group according to the quartiles of the traditional Japanese diet score

	Traditional Japanese diet score			
Food/food group	Q1 (n = 4,630)	Q2 (n = 2,008)	Q3 (n = 3,243)	Q4 (n = 2,879)
White rice	1,055 (22.8)	941 (46.9)	1,737 (53.6)	1,904 (66.1)
Miso soup	625 (13.5)	833 (41.5)	2,001 (61.7)	2,382 (82.7)
Soybean products	804 (17.4)	896 (44.6)	2,148 (66.2)	2,616 (90.9)
Vegetables[1]	286 (6.2)	423 (21.1)	1,406 (42.4)	2,290 (79.5)
Mushrooms	169 (3.7)	268 (13.4)	1,025 (31.6)	2,167 (75.3)
Seaweeds	125 (2.7)	240 (12.0)	968 (29.9)	2,174 (75.5)
Fish and shellfish	391 (8.4)	517 (25.8)	1,409 (43.5)	2,235 (77.6)
High-sodium foods[2]	1,189 (25.7)	970 (48.3)	1,942 (59.9)	2,317 (80.5)
Green tea	1,295 (28.0)	936 (46.6)	1,786 (55.1)	1,988 (69.1)

[1]Including only cooked vegetables (e.g., dishes with lots of vegetables and soups).
[2]Including pickled plums, pickled vegetables, dried fish, salted fish, and fish roe.
Figures are numbers (percentages) unless otherwise stated.
Abbreviations: Q: quartiles

Supplementary Table S2. Number (percentage) of people who achieved the cutoff for each component food/food group according to the quartiles of the modified Japanese diet score

Food/food group	Modified Japanese Diet Score			
	Q1 (n = 3,701)	Q2 (n = 3,850)	Q3 (n = 2,913)	Q4 (n = 2,296)
Whole grains[1]	204 (5.5)	534 (13.9)	532 (18.3)	761 (33.1)
Miso soup	500 (13.5)	1,649 (42.8)	1,801 (61.8)	1,891 (82.4)
Soybean products	488 (13.2)	1,755 (45.6)	2,093 (71.9)	2,128 (92.7)
Vegetables[2]	274 (7.4)	1,465 (38.1)	2,114 (72.6)	2,150 (93.6)
Mushrooms	89 (2.4)	542 (14.1)	1,195 (41.0)	1,803 (78.5)
Seaweeds	85 (2.3)	511 (13.3)	1,125 (38.6)	1,786 (77.8)
Fruits	172 (4.7)	701 (18.2)	1,143 (39.2)	1,630 (71.0)
Fish and shellfish	264 (7.1)	962 (25.0)	1,475 (50.6)	1,851 (80.6)
Milk and dairy products	393 (10.6)	1,334 (34.7)	1,448 (49.7)	1,648 (71.8)
High-sodium foods[3]	1,899 (51.3)	2,148 (55.8)	1,388 (47.7)	907 (39.5)
Green tea	996 (26.9)	1,793 (46.6)	1,607 (55.2)	1,609 (70.1)

[1]Including rice mixed with millet and barley, brown rice, germinated rice, under- or half-milled rice

[2] Including cooked vegetables, raw vegetables, and 100% vegetable juices.

[3] Including pickled plums, pickled vegetables, dried fish, salted fish, and fish roe. Reversal item.

Abbreviations: Q: quartiles

Supplementary Table S3. Summary of existing scoring systems of Japanese diet

	**Japanese Food Guide Spinning Top** [3][14]	**Reduced-Salt Japanese Diet Score** [4]	**Japanese Diet Index** [5][6]	**Japanese Food Score**[8]	**Japanese Healthy Diet Index** [7]
Grains	Grain dishes:<br>5-7 SV	NA	Rice: ≥ median	NA	Rice: ≥ median<br>Bread: <median
Potatoes	Vegetable dishes:<br>5-6 SV	NA	NA	NA	NA
Vegetables		NA	Green and yellow vegetables: ≥median	Vegetables:<br>≥3–4 times/week	Green vegetables: ≥median<br>Red/yellow vegetables: ≥median<br>White vegetables: ≥median<br>Vegetable juices: <median
Seaweeds		NA	Seaweeds: ≥median	Seaweeds:<br>≥3–4 times/week	NA
Mushrooms		NA	NA	Mushrooms:<br>≥3–4 times/week	NA
Pickles	NA	Japanese pickles:<br>≥1 time/day	Pickles: ≥median	Japanese pickles:<br>≥3–4 times/week	NA
Fruits	Fruits: 2 SV	NA	NA	Fruits:<br>≥3–4 times/week	Fruits: ≥median<br>Fruit juices: <median
Pulses[1]	Fish and Meat dishes: 3-5 SV	NA	NA	Boiled beans and tofu:<br>≥3–4 times/week	Tofu: ≥median<br>Fermented beans: ≥median<br>Boiled beans: ≥median<br>Soybean milk: <median
Fish and shellfish		Fish: ≥1 time/2 days	Fish: ≥ median	Fresh fish:<br>≥3–4 times/week	Fish: ≥median
Meats		Meats: ≤2 times/week	Beef and pork: <median	NA	Beef and pork: <median<br>Chicken: <median<br>Ham: <median
Eggs		Eggs: ≤2 eggs/week	NA	NA	NA
Dairy	Milk: 2 SV	NA	NA	NA	Milk: <median<br>Yogurt: <median
Fat and oil	NA	NA	NA	NA	NA

Supplementary Table S3. (continue)

	**Japanese Food Guide Spinning Top** [3][14]	**Reduced-Salt Japanese Diet Score** [4]	**Japanese Diet Index** [5][6]	**Japanese Food Score**[8]	**Japanese Healthy Diet Index** [7]
Confectioneries	Snacks,confections,	NA	NA	NA	NA
Alcohol	and beverages: ≤200 kcal	Occasional drinking: yes	NA	NA	NA
Non-alcohol beverages		NA	Green tea: ≥median<br>Coffee: <median	NA	NA
Soup	NA	Infrequent intake of soup with noodles: yes	Miso soup: ≥ median	NA	Miso soup: ≥ median
Seasonings	NA	Use of low-salt soya sauce: yes	NA	NA	NA
Energy	Energy:<br>2200±200 kcal	NA	NA	NA	NA
Total score	0–70	0–7	0–9	0–7	1-19
Purpose	Promote healthy diet	Extract the beneficial components of the Japanese diet	Characterize the traditional Japanese diet	Characterize the Japanese diet	Assess the quality of the Japanese diet
Population	Children and adults	NIPPON DATA80	Ohsaki cohort	JACC study	Fukushima Health Management Survey
Component selection	NA	NA	Dietary pattern analysis	Dietary pattern analysis	Dietary pattern analysis
Cutoff definition	NA	Association with mortality	Sex-specific median	Common frequency	Median

[1] In the Japanese Food Guide Spinning Top, soybeans and soy products are included in "Fish and Meat dishes", while pulse except soybeans are included in "Vegetable dishes".
